# Potential limitations in systematic review studies assessing the effect of the main intervention for treatment/therapy of COVID-19 patients: An overview

**DOI:** 10.3389/fmed.2022.966632

**Published:** 2022-09-15

**Authors:** Mahsa Mohseni, Hosein Ameri, Morteza Arab-Zozani

**Affiliations:** ^1^Knowledge Utilization Research Centre, Tehran University of Medical Sciences, Tehran, Iran; ^2^Health Policy and Management Research Center, Department of Health Management and Economics, School of Public Health, Shahid Sadoughi University of Medical Sciences, Yazd, Iran; ^3^Social Determinants of Health Research Center, Birjand University of Medical Sciences, Birjand, Iran

**Keywords:** COVID-19, systematic review, limitations, intervention, treatment

## Abstract

**Background:**

Although several studies have assessed the safety, efficacy, and effectiveness of interventions in treating the COVID-19, many of them have limitations that can have an immense impact on their results. This study aims to assess the potential limitations in systematic reviews (SRs) that evaluate the effect of interventions on the treatment of the COVID-19.

**Methods:**

PubMed, Scopus, and Web of Sciences (WOS) databases were searched from inception to January 1, 2022. All systematic reviews investigated the effectiveness, efficacy, safety, and outcome of the main intervention (Favipiravir, Remdesivir, Hydroxychloroquine, Ivermectin, Lopinavir/Ritonavir, or Tocilizumab) for the treatment of COVID-19 patients and reported the potential limitations of the included studies. We assessed the quality of the included studies using the Quality Assessment Tool (QAT) for review articles. We conducted a content analysis and prepared a narrative summary of the limitations.

**Results:**

Forty-six studies were included in this review. Ninety one percent of the included studies scored as strong quality and the remaining (9%) as moderate quality. Only 29.7% of the included systematic reviews have a registered protocol. 26% of the included studies mentioned a funding statement. The main limitations of the included studies were categorized in 10 domains: sample size, heterogeneity, follow-up, treatment, including studies, design, definitions, synthesis, quality, and search.

**Conclusion:**

Various limitations have been reported in all the included studies. Indeed, the existence of limitations in studies can affect their results, therefore, identifying these limitations can help researchers design better studies. As a result, stronger studies with more reliable results will be reported and disseminated. Further research on COVID-19 SRs is essential to improve research quality and also, efficiency among scientists across the world.

## Background

The COVID-19 pandemic began in early 2020 with major health consequences (1). According to live data from Worldometer website, the total number of coronavirus cases and the number of deaths so far is 595,494,252 and 6,455,301, respectively (Tue, 16 Aug 2022). Numerous studies have assessed the effects of the different interventions on the treatment of the COVID-19 patients (2–6). These studies differ in many ways, including the type of treatment, follow-up time, study design, patient type, and disease severity, each of which can have a positive or negative effect on the results of these studies (7).

As the global community eagerly awaits credible scientific solutions for this pandemic, researchers and scientists are under much pressure to identify effective therapeutic and preventive strategies for COVID-19. Also, there are many unknowns, and the massive demand for evidence on the treatment of a novel disease such as COVID-19 may be unintentionally affecting studies’ design and conduct. Furthermore, it may inadvertently affect the peer-review and publication process, leading to significant methodology gaps and overall lower quality evidence on COVID-19. These gaps lead to less-informative studies, loss of precious time, and valuable resources (8).

With the growth of evidence in this area (9), there is a need for studies that report the results of these individual studies in general. Systematic reviews objectively summarize large amounts of information, identifying gaps in medical research, and identifying beneficial or harmful interventions which will be useful for clinicians, researchers, and even for public and policymakers. The value of a systematic review depends on what was done, what was found, and the clarity of reporting (10). The results of a systematic review are influenced by the quality of the primary studies included. Methodologically, poor studies tend to exaggerate the overall estimate of treatment effect and may lead to incorrect inferences (11).

While a need to disseminate information to the medical community and general public was paramount, concerns have been raised regarding the scientific rigor, quality, and limitations in published reports which may potentially effect on the systematic reviews and meta-analysis results (1). In this study, we aim to identify the potential limitations in systematic reviews that evaluated the effect of interventions on the treatment of the COVID-19 which can help to improve and make the result of studies more accurate in the future.

## Methodology

### Protocol and registration

We conducted this overview based on Smith et al. guideline for conducting a systematic review of systematic reviews of healthcare interventions (12). We also followed the PRISMA guideline for reporting the methods and results of this study (13).

### Eligibility criteria

All systematic reviews with available full text and in EN languages investigated the effectiveness, efficacy, safety, and outcome of the main intervention (Favipiravir, Remdesivir, Hydroxychloroquine, Ivermectin, Lopinavir/Ritonavir, or Tocilizumab) for treatment of COVID-19 patients and reported the potential limitation of the study were included.

We exclude articles that are full-text not available or used other treatment options than mentioned drugs. For example, acupuncture or traditional medicine, or supplement therapy. Preprint and without peer review articles also was excluded.

### Information sources and search strategy

We searched PubMed, Scopus, and Web of Sciences (WOS) databases from inception to January 1, 2022, for the keywords COVID-19, “SARS-CoV-2,” “novel coronavirus,” “systematic review,” OR limitation in the title, abstract, or main text of the published article. There was no limitation regarding time or language. We also conducted a manual search in Google Scholar for potential missing articles. In addition to database searches, we screened reference lists of included studies after screening records were retrieved *via* databases and also contacted the corresponding authors of the included studies. The full search strategy for all databases is presented in Supplementary Table 1.

### Selection process

After the search was completed, all retrieved records were imported in EndNote, version X7, and duplicate removed. Two independent reviewers (HA, MM) screened the records based on the title, abstract, and full text. For increasing the agreement between reviewers we piloted a set of 30 studies before the screening. Discrepancies at this stage were resolved by consensus with a third reviewer (MA-Z).

### Data collection process and data items

Two independent reviewers (HA, MM) extracted the data. We designed a data extraction table for this study, which was piloted by two reviewers (5 studies). we extracted the following data: first author name, corresponding authors name and email, Publication year, number of authors, study design, number of included studies in each included systematic review, investigated drug, country, language limitation, time of the search, number of the investigated outcome, sample size, limitations, funding statement, mean age, gender (%), protocol and registration information. Discrepancies at this stage were resolved by consensus with a third reviewer (MA-Z).

### Quality appraisal

Two reviewers (HA and MM) independently assessed the quality of the included studies. We assessed the quality of the included studies using the Quality Assessment Tool (QAT) for review articles developed by healthevidence.org, which was piloted by two reviewers (5 studies) including ten quality criteria. A final review quality rating for each review is assigned: strong (8–10/10), moderate (5–7/10), or weak (1–4/10). Any discrepancies were resolved upon consultation with a third reviewer (MA-Z).

QAT tool available at: https://www.healthevidence.org/our-appraisal-tools.aspx.

### Synthesis of results

For data synthesis, we prepared a table summarizing systematic review information. We also used graphs for presenting some information. We then conducted a content analysis and prepared a narrative summary of the limitations. Two authors (HA, MM) read and reread the results reported in published articles to extract limitations. The coding frame and final categories were developed by 3 authors (HA, MM, and MA-Z) using these data.

## Results

### Study selection

**FIGURE 1:**
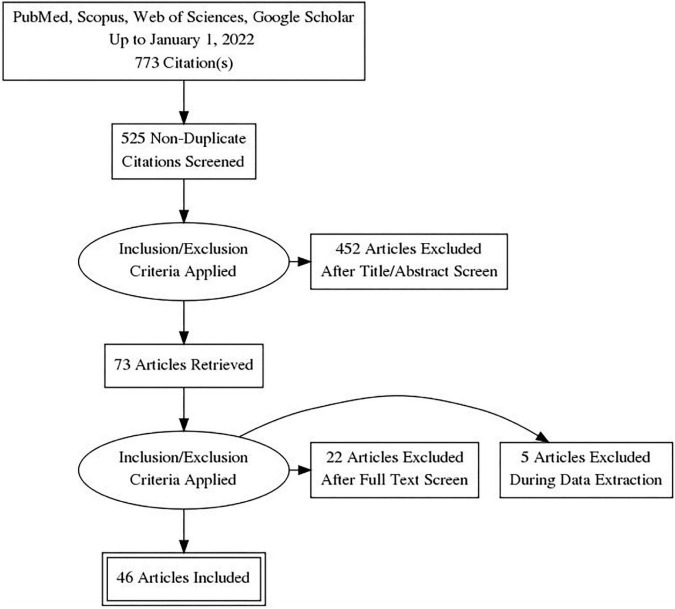
PRISMA flow diagram.

A total of 773 records were retrieved from the database search. After removing duplicates, 525 records were screened by title, abstract, and full-text based on eligibility criteria, of which 46 studies were included in the final review (14–58, 59). Twenty-seven studies were excluded after Full-text screening. The reasons for exclusion were as follows: Protocol (5 records), Preprint (6 records), Full-text not available after contacting the corresponding author (2 records), Not reporting limitation (5 records), and not investigating our target intervention (9 records). The PRISMA flow diagram for the complete study selection process is presented in Figure 1.

### Study characteristics

**FIGURE 2:**
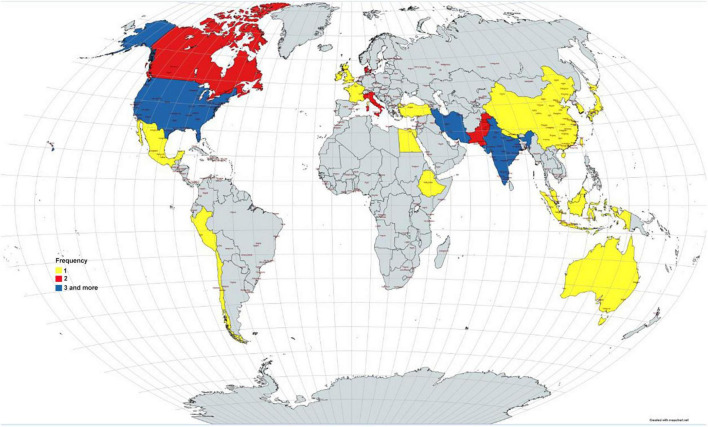
Distribution of the included studies by countries.

**FIGURE 3:**
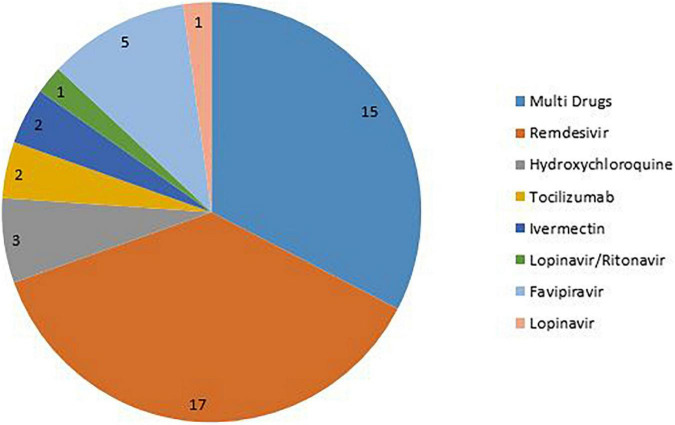
Distribution of the drugs in the included studies.

The number of authors of the included systematic reviews varied between 3 and 58 people. Most studies were from Asia (46%), America (31%), and Europe (17%). Also, by country, most studies were reported from the United States and India (Figure 2). 80.4% of the included systematic review conducted a meta-analysis. The number of included studies in the included systematic reviews varied between 2 and 136. Only 29.7% of the included systematic reviews have a registered protocol. Also, 26% of the included systematic reviews mentioned a funding statement. More details about the characteristics of included systematic reviews are presented in Table 1. The most studied drug in the included studies was Remdesivir (17.37%) (Figure 3).

**TABLE 1:** Summary characteristics of the included studies.

Authors/year	Country	Number of authors	Meta-analysis	Number of included studies	Number of investigated outcome	Overall sample size	Investigated drug	Published protocol	Registration	Funding statement
Abdelrahman et al. (58)	China	7	Yes	136	5	102,345	Multi drugs	No	No	Yes
Al-Abdouh et al. (57)	USA	10	Yes	4	5	7,334	Remdesivir	No	No	No
Angamo et al. (56)	Australia	3	Yes	7	6	3,686	Remdesivir	No	No	No
Ayele Mega et al. (55)	Ethiopia	5	Yes	20	6	6,782	Hydroxychloroquine	No	No	No
Bansal et al. (54)	India	12	Yes	9	4	1,895	Remdesivir	No	No	No
Bartoszko et al. (53)	Canada	40	Yes	11	6	6,701	Multi drugs	No	No	Yes
Bhattacharyya et al. (52)	India	13	Yes	13	7	1,114	Multi drugs	No	No	No
Conti et al. (51)	Italy	11	Yes	47	1	15,196	Tocilizumab	No	Yes	No
Cruciani et al. (50)	Italy	6	Yes	11	4	2,436	Ivermectin	No	Yes	No
Das et al. (49)	India	4	No	12	3	3,543	Hydroxychloroquin	No	No	No
Diaz-Arocutipa et al. (48)	Peru	3	Yes	47	6	13,087	Multi drugs	No	No	No
Elsawah et al. (47)	Egypt	4	Yes	5	9	NR	Lopinavir/ritonavir	No	Yes	No
Fiolet et al. (46)	France	6	Yes	29	1	15,190	Remdesivir	No	Yes	No
Gholamhoseini et al. (45)	Iran	4	Yes	6	4	8,856	Multi drugs	No	No	Yes
Hassanipour et al. (44)	Iran	6	Yes	9	6	825	Remdesivir	No	Yes	No
Hernandez et al. (43)	USA	5	No	23	11	NR	Favipiravir	No	No	Yes
Hussain et al. (42)	UK	5	No	16	1	NR	Multi drugs	No	No	No
Jankelson et al. (41)	USA	5	No	14	4	26,611	Lopinavir	No	No	No
Juul et al. (39)	Denmark	14	Yes	21	3	13,312	Multi drugs	No	No	No
Juul et al. (40)	Denmark	15	Yes	82	6	40,249	Multi drugs	No	No	No
Kaka et al. (38)	USA	7	Yes	5	5	7,767	Multi drugs	No	No	Yes
Kim et al. (37)	Korea	4	Yes	110	8	54,119	Multi drugs	No	Yes	Yes
Kotak et al. (36)	Pakistan	10	Yes	13	6	766	Tocilizumab	No	No	No
Lai et al. (35)	Taiwan	6	Yes	5	9	13,544	Remdesivir	No	Yes	Yes
Manabe et al. (34)	Japan	4	Yes	11	2	1,019	Favipiravir	No	No	Yes
Manzo-Toledo et al. (33)	México	5	Yes	5	2	2,041	Hydroxychloroquine	No	No	No
Murchu et al. (59)	Ireland	6	No	8	6	1,917	Any intervention	No	No	Yes
Okoli et al. (32)	Canada	6	Yes	5	4	13,558	Remdesivir	No	Yes	NR
Özlüşen et al. (31)	Turkey	9	Yes	12	2	1,636	Favipiravir	No	No	NR
Padhy et al. (30)	India	4	Yes	4	4	629	Ivermectin	No	Yes	NR
Piscoya et al. (29)	USA	9	Yes	6	8	2,384	Remdesivir	No	No	Yes
Prakash et al. (28)	India	8	Yes	4	4	405	Favipiravir	No	No	No
Qomara et al. (27)	Indonesia	5	No	15	4	16,339	Multiple drugs	No	No	NR
Rezagholizadeh et al. (26)	Iran	4	Yes	10		6,333	Remdesivir	No	No	No
Roshanshad et al. (25)	Iran	7	No	5	5	1,781	Remdesivir	No	No	No
Santenna et al. (24)	India	7	Yes	15	4	2,342	Remdesivir	No	No	No
Sarfraz et al. (23)	Pakistan	9	Yes	4	9	3,013	Remdesivir	No	No	No
Shrestha et al. (22)	Nepal	6	Yes	9	7	857	Favipiravir	No	No	No
Shrestha et al. (21)	MC*	6	Yes	10	6	5,262	Remdesivir	No	No	No
Siemieniuk et al. (20) A	MC	58	Yes	196	11	76,767	Multiple drugs	Yes	No	Yes
Singh et al. (19)	India	5	Yes	4	4	7,324	Remdesivir	No	Yes	No
Thiruchelvam et al. (18)	Malaysia	4	No	11	2	NR	Remdesivir	No	No	No
Thoguluva Chandrasekar et al. (17)	USA	6	Yes	29	6	5,207	Multiple drugs	No	No	No
Vegivinti et al. (16)	USA	8	Yes	6	4	1,691	Remdesivir	No	No	No
Verdugo-Paiva et al. (15)	Chile	4	Yes	12	9	NR	Multiple drugs	Yes	Yes	No
Wilt et al. (14)	USA	6	No	4	7	2,279	Remdesivir	Yes	No	Yes

*MC, Multi Country.

### Quality appraisal

**FIGURE 4:**
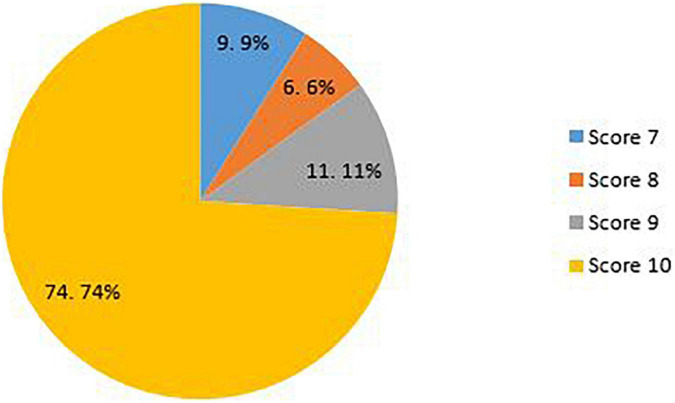
Quality scores of the included studies.

The overall mean quality score of the included studies was 9.5. Overall, 91% of the included studies were scored as strong quality and the remaining (9%) as moderate quality. The overall scores ranged between 7 and 10. About 74% of the included studies had a score of 10, 11% had a score of 9, 6% had a score of 8, and the remaining (9%) had a score of 7 (Figure 4) (for more details about items on the QAT checklist see Supplementary Table 2).

### Results of synthesis

#### Potential limitations of the included studies

Various studies have listed different limitations for the studies, some related to how the systematic review was conducted and some related to the studies included in these systematic reviews. The main limitations of the included studies were categorized in 10 domains: Heterogeneity (4 sub-categories), sample size (2 sub-categories), follow-up (2 sub-categories), treatment (7 sub-categories), included studies (4 sub-categories), design (10sub-categories), definitions (3 sub-categories), synthesis (4 sub-categories), quality (2 sub-categories), and search (4 sub-categories). The highest frequencies reported in the included studies related to the heterogeneity in sample population, small sample size, and database searches.

Heterogeneity in studies has been reported for a variety of reasons, including differences in the sample population regarding age, gender, ethnicity, and racial groups; different level of disease severity in the included patients; different control group; and difference in the investigated outcome. Treatment-related limitations were mostly related to differences regarding the administration of drug, dose, duration of treatment, and different standard protocols and guidelines. Also, there are differences related to discontinuation, combination therapy, and supportive care which obscure the effect of the main treatment.

The studies had several design shortcomings. Many studies have suffered from a lack of randomization, placebo, blinding, and comparator arm. Selection bias, and publication bias, confounding bias were also reported in the studies. Also, different strategies regarding search were another limitation. Different databases, using pre-print and un-published data, limitations on language, and missing some eligible studies were the important limitation in this regard.

More details about the potential limitations of the included systematic reviews are presented in Table 2.

**TABLE 2:** Potential limitation of the included studies.

Category	Sub-category	Frequency
Heterogeneity	•Heterogeneity in sample population	22
	• Heterogeneity in disease severity	9
	•Heterogeneity in control groups	15
	•Heterogeneity in outcome	13
Sample size	•Small sample size	21
	•Different inclusion criteria	12
Follow up	•Short follow up time	14
	•Different follow up time	17
Treatment	•Different standard protocol and guideline	7
	•Different administration of drug	7
	•Different duration of treatment	11
	•Different dose of treatment	8
	•Treatment discontinuation	6
	•Combination therapy	6
	•Supportive care	5
Included studies	•Different type of included studies	7
	•Low number of included studies	14
	•Different level of quality of the included studies	9
	•Short duration of studies	7
Design	•Different design of the included studies	6
	•Randomization	9
	•Placebo	12
	•Blinding	8
	•Single-arm	7
	•Lack of comparator arm	7
	•Different comparator arm	15
	•Selection bias	8
	•Publication bias	6
	•Confounding bias	4
Definition	•Different definition of disease severity	8
	•Different definition of outcomes	9
	•Different definition of ordinal scales	3
Synthesis	•Different meta-analysis approach	5
	•Sub-group analyses	5
	•Lack of important data	6
	•Causality	2
Quality	•Low quality of the included studies	17
	•Low level of evidence	12
Search	•Database search	18
	•Preprint, pre-publish and unpublished study	16
	•Limitation on language	7
	•Missing eligible studies	3

## Discussion

With the spread of the COVID-19 pandemic and has many consequences, the need arose to conduct studies and disseminate their findings (1).

Systematic reviews are a valuable resource in academia and practice. Well-done systematic reviews, which include but are not limited to meta-analyses, offer an efficient way to evaluate a large amount of information for decision-makers in areas of research, policy, and patient care. Systematic reviews can help us know what we know about a topic, and what is not yet known, often to a greater extent than the findings of a single study (60–64). Systemic review studies on the safety and efficacy of COVID-19 have grown in numbers. Regarding the growing number of studies and rapid publication time, there are concerns about accuracy, quality, and limitations. Richard et al. performed a systematic review to evaluate the methodological quality of currently available COVID-19 studies compared to historical controls. This research showed that COVID-19 clinical studies have a shorter time to publication and have lower methodological quality scores than control studies in the same journal (1). We tried to identify the potential limitations of COVID-19 systematic reviews which can improve and make the result of studies more accurate.

The current review examined 46 systematic reviews and all of them were conducted on COVID-19 patients. These studies differ in many aspects, including the type of treatment, follow-up time, study design, patient type, and disease severity. Most of them (80.4%) have conducted meta-analyses. Overall, 91% of the included studies were scored as strong quality, and the rest of them were moderate. The number of studies in the included systematic reviews ranged from 2 to 136.

In this study, we classified the reported limitations into 10 categories and 42 sub-categories. Heterogeneity, sample size, follow-up, treatment, including studies, design, definitions, synthesis, quality, and search are identified as the main limitation of included studies. These limitations were attributed to the included systematic reviews or due to primary studies in these systematic reviews. Among all the limitations, sample population, sample size, and database search were found to be the most-mentioned limitations with frequencies of 22, 21, and 18 in the studies, respectively. It seems that a greater number of limitations could be due to primary studies in the systematic reviews including heterogeneity, small sample size, short follow-up time, and low quality of included studies. Limitations in systematic review studies result from selection of studies, choice of relevant outcomes, methods of analysis, interpretation of heterogeneity, and generalization, application of results, and proper search (65).

Heterogeneity contains four subcategories including differences in the sample population, differences in disease severity of patients, different control group, and different measured outcomes. Differences in the sample population in terms of age, gender, race, and comorbidities in the participants are the most reported limitation. Heterogeneity across the studies may affect the study results (65). For instance, pooling the data of the original articles would be highly difficult due to heterogeneity in the study design and reported outcomes (25), and heterogeneity in disease severity could affect the treatment output.

The small sample size is the second most frequently reported limitation in 21 studies. The number of participants in the included studies was small which could decrease the power of the studies, furthermore comparing the interventions regarding the efficacy would not be incontrovertible. Therefore, the findings need to be interpreted with caution.

Database search is another important item that belongs to the search category and is reported in 18 studies. It may potentially limit access to eligible trials for inclusion and miss some data.

Treatment-related limitations are mostly associated with differences regarding the administration of drugs, dose, duration of treatment, and different standard protocols and guidelines. The lack of uniform guidelines for administering additional treatments and providing supportive care for COVID-19 patients in clinical trials may lead to inaccurate and unreliable outcomes. These limitations can generate confounding bias (36). Also, there are other items belonging to this category such as differences related to discontinuation, combination therapy, and supportive care which obscure the effect of the main treatment.

In addition to the above-mentioned limitations, different follow-up times, low quality of the included studies, pre-publish and unpublished studies, different comparator arms, and heterogeneity in control groups are the other highly reported items. The lack of a comparison/control group can limit the validity of the meta-analysis.

As mentioned, although systematic reviews are considered the gold standard of evidence for clinical decision-making, one should keep in mind that meta-analyses should neither be a replacement for well-designed large-scale randomized studies nor a justification for conducting small underpowered studies (65). As other studies reported, the quality of the methodology and reporting of present COVID-19 SR is far from optimal. In addition, Differences in disease definition and heterogeneity in studies are important factors influencing the results of these studies. Following existing guidelines and proper study design can be one of the factors reducing the limitations of these studies (66, 67). Taken together, poor designs and various limitations of the studies render them ineffective in gaging the full extent of its safety and efficacy and thus warrant further research into the use of interventions in COVID-19 patient treatment. Our study further highlighted the importance of conducting quality studies so that the results can be trusted with more certainty.

### Implications for future research

Our results can be used as a guide for designing and reporting the future studies in this field. Undoubtedly, awareness of the limitations of articles in this field can reduce bias and on the other hand increase the power of studies. Considering these issues helps researchers to report studies in a more integrated way, which can also help readers to better understand the results of studies and prevent the repetition of errors and mistakes or limitations reported in previous studies. It is recommended that researchers interested in research related to COVID-19, as well as those interested in investigating the effectiveness of treatments for this disease, must consider the points mentioned in this study when designing, implementing, and reporting their studies. In addition, respected researchers can design similar studies for other fields related to this disease and report their results. Designing such studies can greatly contribute to evidence-based decision making.

### Strengths and limitations

Although this study is an overview, and the quality appraisal is optional, but the quality of the articles has been evaluated in it, which is one of the strong points of the study. Also, we conducted this overview based on Smith et al. guideline for conducting a systematic review of systematic reviews and report the results of this study based on PRISMA guideline. All the steps of this study were done by two independent reviewers, which reduces errors and increases the power of the study. There are many potential limitations to this overview. First, a literature search was conducted in the three major electronic databases, Scopus, Pubmed, and WOS, but no other databases were searched, as was the “gray” literature. Therefore, additional relevant studies might have been missed. Second, we included all systematic reviews with available full text and in EN languages investigated the effectiveness, efficacy, safety, and outcome of the main intervention (Favipiravir, Remdesivir, Hydroxychloroquine, Ivermectin, Lopinavir/Ritonavir, or Tocilizumab) for treatment of COVID-19 patients. However, there are other interventions for the treatment of this disease, which can be investigated in other studies, but due to the small number of them, they were not included in this study and only the main interventions were used. Third, we excluded articles published in preprint databases due to lack of peer review.

## Data availability statement

The original contributions presented in this study are included in the article/Supplementary material, further inquiries can be directed to the corresponding author.

## Author contributions

MA-Z: idea, design, analyses, writing the first draft, and revising. MM and HA: data extraction, quality appraisal, writing the first draft, and revising. All authors read and approved the final draft before submission.
