# Supplementary material for: Potential limitations in systematic review studies assessing the effect of the main intervention for treatment/therapy of COVID-19 patients: An overview
Source: Front Med (Lausanne). 2022 Sep 15;9:966632. doi: 10.3389/fmed.2022.966632 (PMC9531544; doi:10.3389/fmed.2022.966632)
Supplement: Supplementary file 1 [file Table_1.pdf]

Supplementary table 1: Database search strategy

Database	Query	Number
PubMed	((COVID-19[Title] OR "SARS-CoV-2"[Title] OR "novel coronavirus"[Title]) AND ("systematic review"[Title])) AND (limitation OR limitations)	409
Scopus	(TITLE (covid-19 OR "SARS-CoV-2" OR "novel coronavirus") AND TITLE ("systematic review") AND ALL (limitation OR limitations))	214
WOS	You searched for: TITLE: (COVID-19 OR "SARS-CoV-2" OR "novel coronavirus") AND TITLE: ("systematic review") AND ALL FIELDS: (limitation OR limitations)	122

Supplementary table 2: Quality appraisal of included systematic reviews

Authors/Year	Q1	Q2	Q3	Q4	Q5	Q6	Q7	Q8	Q9	Q10	Score
Abdelrahman et al, 2021	Yes	Yes	Yes	Yes	Yes	No	Yes	Yes	Yes	Yes	9
Al-Abdouh et al, 2021	Yes	Yes	Yes	Yes	Yes	Yes	Yes	Yes	Yes	Yes	10
Angamo et al, 2021	Yes	Yes	Yes	Yes	Yes	Yes	Yes	Yes	Yes	Yes	10
Ayele Mega et al, 2020	Yes	Yes	Yes	Yes	Yes	Yes	Yes	Yes	Yes	Yes	10
Bansal et al, 2020	Yes	Yes	Yes	Yes	Yes	Yes	Yes	Yes	Yes	Yes	10
Bartoszko et al, 2021	Yes	Yes	Yes	Yes	Yes	Yes	Yes	Yes	Yes	Yes	10
Bhattacharyya et al, 2020	Yes	Yes	Yes	Yes	Yes	Yes	Yes	Yes	Yes	Yes	10
Conti et al, 2021	Yes	Yes	Yes	Yes	Yes	Yes	Yes	Yes	Yes	Yes	10
Cruciani et al, 2021	Yes	Yes	Yes	Yes	Yes	Yes	Yes	Yes	Yes	Yes	10
Das et al, 2020	Yes	Yes	Yes	Yes	Yes	Yes	Yes	Yes	Yes	Yes	10
Diaz-Arocutipa et al, 2020	Yes	Yes	Yes	Yes	Yes	Yes	Yes	Yes	Yes	Yes	10
Elsawah et al, 2020	Yes	Yes	Yes	Yes	Yes	Yes	Yes	Yes	Yes	Yes	10
Fiolet et al, 2021	Yes	Yes	Yes	Yes	Yes	Yes	Yes	Yes	Yes	Yes	10
Gholamhoseini et al, 2021	Yes	Yes	Yes	Yes	Yes	Yes	Yes	Yes	Yes	Yes	10
Hassanipour et al, 2021	Yes	Yes	Yes	Yes	Yes	Yes	Yes	Yes	Yes	Yes	10
Hernandez et al, 2020	Yes	Yes	Yes	Yes	Yes	Yes	Yes	Yes	Yes	Yes	10
Hussain et al, 2021	Yes	Yes	Yes	Yes	Yes	Yes	Yes	Yes	Yes	Yes	10
Jankelson et al, 2020	Yes	Yes	Yes	Yes	Yes	No	No	Yes	No	Yes	8
Juul et al, 2020	Yes	Yes	Yes	Yes	Yes	Yes	Yes	Yes	Yes	Yes	10
Juul et al, 2021	Yes	Yes	Yes	Yes	Yes	Yes	Yes	Yes	Yes	Yes	10
Kaka et al, 2020	Yes	Yes	Yes	Yes	Yes	Yes	Yes	Yes	Yes	Yes	10
Kim et al, 2020	Yes	Yes	Yes	Yes	Yes	Yes	Yes	Yes	Yes	Yes	10
Kotak et al, 2020	Yes	Yes	Yes	Yes	Yes	Yes	Yes	Yes	Yes	Yes	10
Lai et al, 2021	Yes	Yes	Yes	Yes	Yes	Yes	Yes	Yes	Yes	Yes	10
Manabe et al, 2021	Yes	Yes	Yes	Yes	Yes	Yes	Yes	Yes	Yes	Yes	10
Manzo-Toledo et al, 2021	Yes	Yes	Yes	Yes	Yes	Yes	Yes	No	Yes	Yes	9
Murchu et al, 2021	Yes	Yes	Yes	Yes	Yes	Yes	Yes	No	No	Yes	8
Okoli et al, 2021	Yes	Yes	Yes	Yes	Yes	Yes	Yes	Yes	Yes	Yes	10
Özlüşen et al, 2021	Yes	Yes	Yes	Yes	Yes	Yes	Yes	Yes	Yes	Yes	10
Padhy et al, 2020	Yes	Yes	Yes	Yes	Yes	Yes	Yes	Yes	Yes	Yes	10
Piscoya et al, 2020	Yes	No	Yes	Yes	Yes	Yes	Yes	Yes	Yes	Yes	9
Prakash et al, 2020	Yes	Yes	Yes	Yes	Yes	Yes	Yes	Yes	Yes	Yes	10
Qomara et al, 2021	Yes	Yes	Yes	Yes	Yes	Yes	Yes	No	No	Yes	8
Rezagholizadeh et al, 2021	Yes	Yes	Yes	Yes	Yes	Yes	Yes	Yes	Yes	Yes	10
Roshanshad et al, 2020	Yes	Yes	Yes	No	Yes	Yes	Yes	No	No	Yes	7
Santenna et al, 2021	Yes	Yes	Yes	Yes	Yes	Yes	No	Yes	Yes	Yes	9
Sarfraz et al, 2020	Yes	Yes	Yes	Yes	Yes	No	No	Yes	Yes	No	7
Shrestha et al, 2020a	Yes	Yes	Yes	Yes	Yes	Yes	Yes	Yes	Yes	Yes	10
Shrestha et al, 2020b	Yes	Yes	Yes	Yes	Yes	Yes	Yes	Yes	Yes	Yes	10
Siemieniuk et al, 2020	Yes	Yes	Yes	Yes	Yes	Yes	Yes	Yes	Yes	Yes	10
Singh et al, 2020	Yes	Yes	Yes	Yes	Yes	Yes	Yes	Yes	Yes	Yes	10
Thiruchelvam et al, 2021	Yes	Yes	Yes	Yes	Yes	No	Yes	No	No	Yes	7
Thoguluva Chandrasekar et al, 2020	Yes	Yes	Yes	Yes	Yes	Yes	Yes	Yes	Yes	Yes	10
Vegivinti et al, 2020	Yes	Yes	Yes	Yes	Yes	Yes	Yes	Yes	Yes	Yes	10
Verdugo-Paiva et al, 2020	Yes	Yes	Yes	Yes	Yes	Yes	Yes	Yes	Yes	No	9
Wilt et al, 2020	Yes	Yes	Yes	Yes	Yes	Yes	Yes	No	No	No	7
Total	46	45	46	45	46	42	43	40	40	43	437
